# Using self-paced reading in research with heritage speakers: a role for reading skill in the online processing of Spanish verb argument specifications

**DOI:** 10.3389/fpsyg.2023.1056561

**Published:** 2023-05-03

**Authors:** Jill Jegerski, Gregory D. Keating

**Affiliations:** ^1^Department of Spanish and Portuguese, University of Illinois, Urbana, IL, United States; ^2^Department of Linguistics and Asian/Middle Eastern Languages, San Diego State University, San Diego, CA, United States

**Keywords:** heritage speakers, Spanish (in the U.S.), self-paced reading (SPR), online methods, verb transitivity, reading skill

## Abstract

Relatively little is known about how heritage speakers process language in real time, despite recent calls for the use of online methods such as self-paced reading, eyetracking, and ERPs (event-related potentials) in research on this early bilingual population. The present study addressed this gap with an empirical study of the online processing of heritage speakers of Spanish in the U.S. using self-paced reading, which is the online method that is most accessible to a wide body of researchers because it does not require specialized equipment. The processing target was related to the online integration of verb argument specifications, which was chosen because it does not involve ungrammatical sentences and therefore may be less likely to involve metalinguistic knowledge and less likely to put heritage speakers at a disadvantage than measures that rely on the recognition of grammatical errors. More specifically, this study examined an effect that occurs when a noun phrase appears after an intransitive verb, which can cause processing difficulty relative to a comparison condition in which the verb is transitive. The participants were 58 heritage speakers of Spanish and a comparison group of 16 first-generation immigrants raised in Spanish-speaking countries. Both groups showed the expected transitivity effect on the post-verbal noun phrase during self-paced reading, but the heritage speaker group also showed a spillover effect on the post-critical region. Among the heritage speakers, these effects were associated with lower self-ratings for reading skill in Spanish and with slower average reading speed during the experiment. Three theoretical accounts of the apparent susceptibility to spillover effects among heritage speakers are proposed: that it is a characteristic of shallow processing, that it is due to underdeveloped reading skill, and that it is an artifact of the self-paced reading method. The latter two possibilities are especially consistent with a role for reading skill in these results.

## 1. Introduction

Heritage speakers are bilingual users of a minority or community language that they have been exposed to at home from birth, but which they have typically had limited opportunities to develop, especially with language skills that are associated with formal education. For instance, heritage speakers often have underdeveloped literacy, metalinguistic skills, and formal register, as compared to their own language skills in the majority language and relative to their counterparts raised in other countries with the same L1 as a majority language (Carreira and Kagan, 2011). This difference between populations of language users raises the question of whether heritage speakers may respond differently to research methods that rely on those skills than other participant groups to which they are frequently compared in empirical studies, including more prototypical L1 users with formal education in the language and classroom-instructed adult second language (L2) learners. A number of scholars have therefore advocated for a move away from experimental tasks that rely on metalinguistic knowledge (Benmamoun et al., 2010) and toward online (i.e., real-time) methods like self-paced reading, eyetracking, and ERPs (event-related potentials; Bolger and Zapata, 2011; Jegerski, 2018a; Jegerski and Sekerina, 2021). One key advantage of online methods is that they record data in real time, while the participant is engaged in a language-related experimental task such as reading or listening, in which words are often processed in as little as 250 milliseconds. These time constraints are thought to reduce the application of metalinguistic knowledge (Montrul et al., 2008; Carreira and Kagan, 2011), so there may be less room for distortion in the data.

The present study employed the self-paced reading method because it is more accessible to a wider body of researchers than other online methods in terms of cost and the level of technical knowledge required. The focus was on a previously-documented processing phenomenon related to the online integration of verb argument specifications and for which the stimulus sentences are all grammatical, so the effect may be less likely to involve metalinguistic knowledge than processing effects that occur with ungrammatical sentences. The use of a reading-based method could be a potential concern with heritage speakers, given that they tend to have underdeveloped literacy, but over 90% of heritage speakers of Spanish in the U.S. have at least an intermediate reading level (Carreira and Kagan, 2011), which would likely be enough for self-paced reading. Indeed, previous research had successfully used self-paced reading with this population on several occasions prior to the present study (Foote, 2011; Jegerski et al., 2016; Keating et al., 2016; Jegerski, 2018b,c).

Like most previous work on heritage speakers using self-paced reading and other online methods (to be discussed in greater detail in the following sections), the present study examined the processing of different sources of linguistic information in real time. The fundamental question driving such research is whether grammatical details such as gender agreement or relative clause attachment are quickly accessed and integrated into an underlying representation of the segment of language that is being comprehended. Some theories propose that such grammatical details are sometimes overlooked during real-time processing, especially among less proficient language users such as adult second language learners (e.g., Clahsen and Felser, 2006; Christianson, 2016). The limited research on online processing among heritage speakers has mostly taken a similar approach, often with the inclusion of some type of comparison group of more prototypical L1 users as a point of reference for sensitivity to the linguistic target during processing (e.g., Sekerina and Trueswell, 2011; Jegerski, 2018c). More recently, some researchers have begun to examine within-group variability in processing among heritage speakers in relation to individual difference variables such as proficiency (Bice and Kroll, 2021) and age of acquisition of the L2 majority language (Keating, 2022). The present study took a combined approach, including both a comparison group of L1 users of Spanish who were first-generation immigrants (to approximate the language that heritage speakers received as input while growing up in the U.S.; Polinsky and Scontras, 2020) and an analysis of within-group variability among heritage speakers.

## 2. Literature review

### 2.1. The processing of verb transitivity in Spanish

The present study examined a processing effect that is known to occur in both English and Spanish and which has been observed with both self-paced reading (Berghoff, 2020) and eyetracking (Staub, 2007). In sentences like (1) below, processing difficulty typically occurs on the first post-verbal noun phrase *the veterinarian* when the verb it follows is intransitive as in (1b), compared to when the verb is transitive as in (1a).

(1)

a. After the dog scratched the veterinarian took off the muzzle. TRANSITIVE.

b. After the dog struggled the veterinarian took off the muzzle. INTRANSITIVE.

The observed processing difficulty is thought to arise from a conflict between the processing principle of Late Closure, which is a preference to incorporate each word into the current phrase whenever possible rather than to initiate a new clause (Frazier and Fodor, 1978), and the argument specifications of the intransitive verb, which do not allow such a structure. In other words, there is a tendency to process the post-verbal noun phrase as a direct object, but this is not possible when the verb in question is intransitive. A similar processing effect has been observed when the verb is transitive but the post-verbal noun phrase is semantically implausible as a direct object, as in *As the men drank the song*… versus *As the men drank the beer*… (Roberts and Felser, 2011). There is evidence these effects can be more robust among slower readers (Roberts and Felser, 2011; Jegerski, 2012), possibly because slower reading may lead to more incremental processing than faster reading (Roberts and Felser, 2011, p. 323), and also that L1 users do not always show an online plausibility effect during reading (Roberts and Felser, 2011).

From the perspective of acquisition, language users who exhibit the aforementioned effect must have acquired the relevant verb argument specifications and have the ability to apply them efficiently during online processing, along with the processing principle of Late Closure. Otherwise, the two would not be in conflict and there would not be an increase in processing difficulty.

### 2.2. The processing of verb transitivity in bilingual populations

To our knowledge, no prior investigation has examined the verb transitivity effect targeted in the present study among heritage speakers, so it is not known whether they are sensitive to this type of information during online processing. There have been two related studies that included early bilinguals (Berghoff, 2020; McCormick, 2020), but the target languages were not minoritized, so the participants were not heritage speakers. The first of these two prior investigations was Berghoff’s (2020) self-paced reading study, in which the bilingual participants were L1 Afrikaans speakers tested in their L2, English, in South Africa. Another difference between that investigation and the present one was that the linguistic stimuli for that study manipulated the semantic plausibility of the post-verbal noun phrase as an object (e.g., *As the men drank the song*… versus *As the men drank the beer*…; Roberts and Felser, 2011) to create conflict with the processing principle of Late Closure, whereas the present study manipulated the transitivity of the verb. Berghoff (2020) compared the early Afrikaans-English bilinguals to late English-Afrikaans bilinguals and both groups showed the expected reading time increases on a post-verbal noun phrase that was implausible as a direct object of the verb versus a noun phrase that was a plausible object. In other words, they appeared to rapidly integrate verb argument specifications during online processing.

The second related investigation was McCormick (2020), which included simultaneous (2 L1) Catalan-Spanish bilinguals in Spain. This self-paced reading study used Spanish stimuli that were nearly identical to those for the present study (both taken from Jegerski, 2012), so verb transitivity was manipulated. The expected reading time increase was observed when a post-verbal noun phrase followed an intransitive verb rather than a transitive one. Additionally, McCormick (2020) also employed a cognitive control engagement paradigm, in which the self-paced reading stimuli alternated with trials in a flanker task,^1^ and found that the verb transitivity effect was diminished when the stimulus was read after an incongruent flanker trial as opposed to a congruent trial. This appeared to be a Gratton effect (Gratton et al., 1992), in which there is prolonged cognitive engagement following stimuli with conflict, so the researcher concluded that the same cognitive control mechanisms were used to resolve conflict in the intransitive verb stimuli as in the flanker task.

Other relevant previous research comes from adult L2 acquisition, where the focus is on the L2 of late bilinguals. In this area, at least five studies have reported apparent processing difficulty on a post-verbal noun in similar stimuli, although the effect was not always due to verb transitivity and in some cases it was an incidental finding rather than the focus of the investigation. Jegerski (2012) observed the same effect targeted in the present study in the self-paced reading times of adult L2 learners of Spanish and a monolingual L1 comparison group, which suggests that L2 learners can acquire verb subcategorization information and apply it efficiently during online processing. On the other hand, the L2 participants in that study were of very high proficiency and two subsequent studies with L2 learners at a lower proficiency level found that verb transitivity did not affect their processing of the post-verbal noun phrase (Brothers et al., 2021: eyetracking study of L2 English; McCormick, 2020: self-paced reading study of L2 Spanish), so it appears that a certain level of language proficiency is necessary to successfully acquire and integrate verb subcategorization specifications during the processing of post-verbal nouns. Frenck-Mestre and Pynte (1997) observed a similar lack of transitivity effect among a group of L2 users of English that were of higher proficiency than in Brothers et al. (2021) and probably lower than in Jegerski (2012), but the L1 group in that study also showed only a marginal effect (*p* = 0.09) and the L2 group did show a nonsignificant numerical difference in the expected direction, so it seems possible that this eyetracking study may have been underpowered with only 16 participants in each group, L1 and L2. Finally, Roberts and Felser (2011) observed that advanced proficiency L2 learners showed even more robust processing effects than L1 users, but their stimuli manipulated the semantic plausibility of the post-verbal noun phrase as a direct object of the verb rather than verb transitivity. Roberts and Felser (2011) therefore concluded that L2 learners are overly sensitive to semantics during processing and argued that this is a compensatory strategy to make up for a lack of sensitivity to syntax and morphosyntax, in line with the theory of Clahsen and Felser (2006).

Thus, early bilinguals appear to integrate verb argument specifications during online processing in their L1 and L2, although the participants in previous work were speakers of two mainstream languages rather than heritage speakers of a minoritized language. There is also evidence that such effects may be related to cognitive control mechanisms, in addition to the linguistic knowledge and processing strategy (Frazier and Fodor, 1978) involved. Late bilinguals have exhibited similar verb transitivity effects while processing their L2, but this appears to require a relatively high level of proficiency. Based on this existing body of evidence, a reasonable expectation is that heritage speakers would also show online verb transitivity effects in their home language, assuming they have acquired a sufficiently high level of proficiency. Hence, our prediction for the present study was that at least some heritage speakers of Spanish would show a verb transitivity effect during self-paced reading and that the effect might vary according to Spanish proficiency level as an individual difference variable. Such an observation would contribute to our knowledge of areas of resilience among heritage speakers, in line with a recent call to broaden the prevailing research focus beyond areas of vulnerability and divergence (Polinsky and Scontras, 2020).

### 2.3. Online methods in research with heritage speakers

As outlined in the introduction to this article, a number of scholars have called for research on heritage speakers using online (i.e., real-time) psycholinguistic methods (Bolger and Zapata, 2011; Jegerski, 2018a; Jegerski and Sekerina, 2021). To date, only a limited number of studies have been conducted using these techniques, but there are enough to suggest that the methods can be useful in work on heritage speakers.

Of the three most common online research methods, self-paced reading, eyetracking, and ERPs, self-paced reading is the most accessible to a wide body of researchers because it does not require specialized equipment, it is much less expensive than the other two methods, and it does not require as much technical training. Nevertheless, it does require dedicated software, extensive knowledge of materials design (see Keating and Jegerski, 2015, for more information), and knowledge of the current approaches to statistical analysis, which have become more complex over time. At least five research studies using self-paced reading with heritage speakers have been published to date (i.e., prior to the publication of this research topic in *Frontiers in Psychology*). One early example is Foote’s (2011) investigation of the processing of agreement by heritage speakers of Spanish, which revealed online sensitivity to both gender and number agreement that was similar to that of a comparison group of more prototypical L1 users who were raised abroad with Spanish as a majority language. Additional previous research with self-paced reading has examined relative clause attachment (Jegerski et al., 2016; Jegerski, 2018b), pronominal reference (Keating et al., 2016), and differential object marking (Jegerski, 2018c), all in heritage Spanish.

Eyetracking has also been used in several studies of heritage speakers, both with text and with the visual world paradigm (in which the linguistic stimuli are auditory and the visual stimuli are images or physical objects). For instance, Sekerina and Trueswell’s (2011) visual world experiment showed that heritage speakers of Russian were slower than monolinguals to integrate word order and visual context in the processing of contrastive focus during auditory processing. In another example using the visual world eyetracking paradigm, Jegerski and Sekerina (2020) observed that heritage speakers of Spanish showed similar online sensitivity to the object marker “a” in auditory questions as more prototypical L1 users raised abroad, even though the heritage speakers were less accurate in their offline responses to the questions. Finally, the results of Fuchs (2021) visual world eyetracking study suggested that heritage speakers of Spanish were able to use grammatical gender for predictive processing of auditory stimuli, similar to a monolingual comparison group.

In a study that employed eyetracking with written stimulus sentences rather than auditory stimuli, Keating (2022) compared the processing of Spanish gender agreement among heritage speakers who had acquired their two languages simultaneously to those who had acquired them sequentially and found that online sensitivity occurred earlier in the eye movement record for the sequential bilinguals, who had longer exposure to just Spanish before beginning to acquire the majority language, English. Parshina et al. (2022) also used eyetracking with text to show that heritage speakers of Russian could predict lexical and morphosyntactic information for upcoming words while reading and that this ability appeared to correlate with literacy experience in Russian. Lastly, Parshina et al. (2021) used eyetracking to document some general tendencies in the reading behavior of heritage speakers of Russian as compared to monolingual readers, more specifically, that they read more slowly, that they were less likely to skip words (which is a normal part of fluent reading), and that they were more likely to reread than the comparison group.

Finally, we are aware of three studies that have employed the ERP method, all with heritage speakers of Spanish. First, Martohardjono et al. (2017) observed that heritage speakers, like a comparison group of more prototypical L1 users raised in Spanish-speaking countries, exhibited the expected P600 and N400 waveforms in response to different types of syntactic anomalies. Second, Rossi (2021) found individual variation in the ERP responses of heritage speakers to gender and number violations. Specifically, the group as a whole did not show sensitivity to gender and number, but a subset of participants did show the expected P600 waveform response, while others showed an N400, which is typically observed in response to semantic anomalies rather than morphosyntactic ones. Finally, Bice and Kroll (2021) observed smaller P600 and N400 responses among heritage speakers than with monolinguals and the researchers also found that variability in the heritage speakers was related to proficiency, whereas with the monolinguals the main factor was working memory.

To summarize, a number of prior empirical investigations have employed online methods in research with heritage speakers. Although a number of scholars working with heritage languages have proposed that online methods can and should be used with this population because they tend to be less metalinguistic than more traditional techniques (e.g., Bolger and Zapata, 2011; Jegerski, 2018a; Jegerski and Sekerina, 2021), empirical experimentation is a critical piece that can establish support for such claims (e.g., Martohardjono et al., 2017). Hence, it is important to note that the outcomes of these studies have been generally positive with regard to methodology, meaning that heritage speakers have often shown the effects that would be expected in research on other populations of language users. Some of the cited researchers have even concluded that online methods are especially appropriate because they can reveal a higher level of heritage language ability than would be evident in other measures (e.g., Martohardjono et al., 2017). On the other hand, an additional goal of this line of work is to determine to what extent there may be special considerations that should guide work using online methods with heritage speakers. This is particularly true with reading-based methods, as pointed out by some of the researchers cited above (Jegerski, 2018b; Parshina et al., 2021, 2022), because literacy tends to be underdeveloped among heritage speakers (Carreira and Kagan, 2011).

Given this background and the goals of this research topic in *Frontiers in Psychology*, the objective of the present study was to contribute to the very limited but growing body of work on heritage speakers using online methods, with particular attention to the research methodology and its effectiveness with this participant population. More specifically, the current investigation was a self-paced reading study of the processing of verb transitivity (as outlined above) among heritage speakers of Spanish and a comparison group of more prototypical L1 users raised with Spanish as a majority language and who, as first-generation immigrants to the U.S., were also bilingual. We also examined the role of several background variables that tend to vary between heritage speakers and more prototypical L1 users, that pertain to reading specifically, or that are of general interest with heritage speakers: self-rated reading ability in Spanish, average reading speed during the experimental self-paced reading task, age of acquisition of English, Spanish proficiency test score, and self-reported current exposure to Spanish.

## 3. Method

### 3.1. Participants

The two participant groups for this study were selected to (1) represent U.S. heritage speakers of Spanish with a range of key individual difference variables such as proficiency, age of onset of bilingualism, and measures of reading skill and (2) to compare the sentence processing of heritage speakers with that of L1 users who would have provided them with input while they were growing up in the U.S., meaning first generation immigrants (the “appropriate baseline” for heritage speakers, as per Polinsky and Scontras, 2020, p. 5). The primary group of interest was comprised of 58 heritage speakers of Spanish who were all early Spanish-English bilinguals that were exposed to Mexican Spanish from birth. The comparison group was comprised of 16 immigrants who were also native speakers of Mexican Spanish and who had acquired the language as children in Mexico, where they received formal education. The participants in the comparison group were also bilingual because they knew English, but they had not begun to acquire the language until at least age 12. All participants were recruited on the campus of a large public university in non-borderland Texas and tested in person. More detailed participant background information is provided in Table 1, where it can be seen that the two groups differed with regard to the individual difference variables of Spanish proficiency test score, self-ratings for speaking, understanding, and reading skills in Spanish and English, and estimated relative exposure to both languages. In addition, as seen in the standard deviations in Table 1, there was variability within each group with regard to these measures and greater variability among the heritage speakers, which is common with this population and was intentional in this study because of the analysis of individual differences.

**Table 1:** Language background information.

	Heritage speakers (*n* = 58)			Comparison group (*n* = 16)		
	*M*	*SD*	Range	*M*	*SD*	range
Age of acquisition						
English	3.12	2.63	0–8	14.00	1.55	12–16
Spanish	0.17	0.60	0–3	0.00	0.00	0
DELE score	35.81	6.80	24–47	45.56	3.42	38–49
Self-rating of skills: English						
Understanding	9.43	0.80	7–10	8.31	1.08	7–10
Speaking	9.36	0.87	6–10	7.75	1.13	5–10
Reading	9.34	0.85	7–10	8.19	0.75	7–9
Self-rating of skills: Spanish						
Understanding	8.49	1.66	1–10	9.63	0.50	9–10
Speaking	7.89	1.60	1–10	9.44	0.63	8–10
Reading	7.51	1.67	3–10	9.44	0.63	8–10
Current exposure						
English	64.12%	16.87	25–100	43.75%	23.92	3–80
Spanish	35.21%	17.04	0–75	55.31%	23.92	20–97
Average reading speed	995 ms	249	675–1836	886 ms	200	550–1246
Age	22.91	9.31	18–60	25.75	5.73	18–38

The maximum DELE score was 50 and the maximum for self-rated proficiency was 10.

### 3.2. Materials

An example of the self-paced reading stimuli can be seen in (2) below, where the slashes indicate how the sentence was segmented into phrases. The 20 sentences for the present experiment were based on those employed in two prior self-paced reading studies (Jegerski, 2012; McCormick, 2020), so they were known to elicit the desired processing effects in monolingual native speakers, 2 L1 Catalan-Spanish bilinguals, and late L2 learners of Spanish with high proficiency. As described in the literature review above, sentences such as those in (2) below are typically associated with longer reading times on the first post-verbal noun phrase *el violín* “the violin” when the verb it follows is intransitive as in (2b), compared to when the verb is transitive as in (2a).

(2) *Stimulus for Self-Paced Reading.*

a. Mientras el maestro/tocaba /el violín/resonaba/por todo el salón. TRANSITIVE.

“While the maestro/played /the violin/resonated/throughout the hall.”

b. Mientras el maestro/descansaba /el violín/resonaba/por todo el salón. INTRANSITIVE.

“While the maestro/took a break/the violin/resonated/throughout the hall.”

The observed processing difficulty is thought to arise when the comprehender integrates both the processing principle of Late Closure, which is a preference to incorporate each word into the current phrase whenever possible rather than to initiate a new clause (Frazier and Fodor, 1978), and the argument specifications of the intransitive verb, which do not allow such a structure. In other words, there is a tendency to process the post-verbal noun phrase as a direct object, but this is not possible when the verb in question is intransitive, so there is a conflict that needs to be resolved during processing.

Each of the 20 stimuli appeared in two conditions, transitive and intransitive. The transitive and intransitive verbs were similar to each other in terms of frequency (Davies, 2005), according to an independent-samples *t*-test: *t*(38) = 0.048, *p* = 0.962. This was only to ensure ease of lexical access; reading times for the different verbs were not compared to each other in any of the statistical analyses. The critical region of interest for which data were analyzed was the post-verbal noun phrase, which was identical in both conditions, so all relevant linguistic variables were controlled. The post-critical region was also identical in both the transitive and intransitive conditions. Each sentence began with a subordinating conjunction such as *mientras* “while,” *antes de que* “before,” or *cuando* “when.”

The stimulus materials design and counterbalancing were as recommended by Jegerski (2014) and Keating and Jegerski (2015). Two counterbalanced presentation lists were created with 10 critical sentences in each condition and each sentence appearing only once in any condition per list. The 20 target stimuli were combined with 140 total distractors and fillers. The distractors were 40 stimuli for another experiment that focused on relative clause attachment (Jegerski, 2018b), as exemplified in (3) below, and the fillers were non-experimental sentences that did not target or manipulate any particular linguistic form. The filler and distractor sentences varied in terms of length, but most were complex with two clauses. The stimuli were presented in pseudo-random order such that no two sentences of the same type appeared in succession.

(3) *Distractor for Self-Paced Reading.*

El jurado / consultó/con la abogada/del acusado/que estaba parada.

“The jury/consulted/ with the lawyer/of the defendant/who was standing.”

Beyond the self-paced reading task, the materials included a Spanish proficiency test and a background questionnaire. The proficiency test was one that has been used for at least 20 years in research on the acquisition of Spanish, starting with Montrul and Slabakova (2003), and which has more recently been shown to correlate with other measures of proficiency such as elicited imitation among heritage speakers (Solon et al., 2022). It is a modified version of the written DELE (*Diploma de español como lengua extranjera* “Diploma of Spanish as a Foreign Language”) with 50 items targeting grammar and vocabulary and for which the maximum score is 50.

The questionnaire was the Language Experience and Proficiency Questionnaire (LEAP-Q; Marian et al., 2007), which included the key individual difference variables of age of acquisition of English, self-reported current exposure to Spanish (“What percentage of the time are you currently and on average exposed to each language?”), and self-rated reading ability in Spanish, plus the additional participant background information that is reported in Table 1.

### 3.3. Procedure

The self-paced reading stimuli were presented in a left-to-right, non-cumulative format using SuperLab (Cedrus Corporation, 2007). Each trial started with a “+” cue symbol that appeared at the leftmost edge of where the first segment of the stimulus sentence would appear; this was to encourage participants to look at the stimulus right away, beginning with the first word, rather than at other parts of the display. Words were masked with dashes but spaces and punctuation remained visible. Participants used a button on a response pad to proceed through each segment of a stimulus sentence at their own pace. Each segment contained one or more words, as illustrated above in (2).

After all segments of a stimulus sentence had been read, a subsequent display screen showed a binary choice comprehension question. As seen in Example (4) below, which followed the example stimulus in (2) above, the post-stimulus questions targeted the meaning of the sentence rather than the participant’s interpretation of a specific linguistic form (and this is why we refer to them as *comprehension* questions rather than *interpretation* questions). Participants responded to the questions using two keys on a Cedrus RB-730 response pad marked with the letters “A” and “B,” which were on the left and right sides of the response pad, respectively. The target responses were counterbalanced such that half were “A” and half were “B” and they were also randomized, to avoid the effects of handedness or other biases.

(4) *Post-Stimulus Comprehension Question.*

¿Dónde puede estar este músico?

a. En un parque.

b. En un teatro.

“Where might this musician be?”

“a. In a park.”

“b. In a theater.”

Detailed instructions and five practice items were presented prior to the experimental block. Participants were told that the test targeted reading comprehension in Spanish and no feedback was provided during the experiment. An optional 10-min break was offered when the participant had read half of the 160 total sentences included in the self-paced reading. Each participant completed all the experimental tasks in a single session lasting 60 to 90 min, including the background questionnaire, the self-paced reading, and the proficiency test, in that order. Participants were paid for their time.

### 3.4. Statistical analysis

All data were analyzed via mixed effects linear and logistic regression using R (R Development Core Team, 2019) with the *lme4* package (Bates et al., 2015). The models included verb transitivity, group, and the transitivity × group interaction as fixed effects, plus subject and item as crossed random effects. Because the two participant groups were not exactly matched for age (heritage speakers m = 22.9, comparison group m = 25.8; see Table 1) and age can affect reading times, it was included as a covariate in all of the statistical models. Deviation coding was used to obtain main effects. Logit models were used with binary choice comprehension accuracy data (Jaeger, 2008). Following current procedure in psycholinguistics for identifying the maximal random effect structure appropriate for the sample (Barr, 2013), each model was first run with the maximal random effect structure, then in cases where that model did not converge, it was incrementally simplified to identify the maximal effect structure that still converged. In the case of interactions in the primary models, pairwise comparisons were examined using the *emmeans* package with the Bonferroni correction (Lenth et al., 2018). R code with the final random effects structure for each of the main analyses can be found under the corresponding output tables. *p* values were obtained using Satterthwaite’s approximation for degrees of freedom with the *lmerTest* package for R (Kuznetsova et al., 2014). Prior to statistical analysis, outlying reading times of less than 100 milliseconds were eliminated because they are more likely to represent errors (e.g., premature button presses in this study) than true linguistic processing (Rayner, 1998) and those beyond 5000 milliseconds were trimmed to the cutoff value, which affected 0.39 and 0.64% of the data, respectively. Response times were also log transformed to reduce the positive skew. Alpha was set at 0.05 for all analyses and *p* values of 0.05 to 0.10 would have been considered to be marginally significant in order to reduce the chance of a Type II error (i.e., a false negative; Larson-Hall, 2010), although none of the analyses for this study yielded any such marginal *p* values.

## 4. Results

Mean accuracy proportions and response times for the post-stimulus comprehension questions, by group and transitivity, can be found in Table 2. The statistical analyses for these data are reported in Table 3. Accuracy was high overall (heritage speakers: *M* = 0.892, SD = 0.311; comparison group: *M* = 0.934, SD = 0.248), which indicates that participants generally paid attention while reading, although the heritage speakers were less accurate overall than the comparison group. There was no effect of transitivity or interaction between the two factors in the analysis of the accuracy data. There was also no effect of age. In addition, the analysis of the response times for the post-stimulus comprehension questions also revealed a main effect of group, in which the heritage group was slower to respond than was the comparison group. There was also an effect of age, which reflected longer response times among older participants. There was no effect of transitivity and no interaction of transitivity with group.

**Table 2:** Comprehension question accuracy and response times (SDs in parenthesis).

	Heritage speakers	Comparison group
*Accuracy*		
Transitive	0.89 (0.31)	0.94 (0.24)
Intransitive	0.89 (0.31)	0.93 (0.25)
*Response times*		
Transitive	3839 (1026)	3547 (1064)
Intransitive	3895 (1022)	3664 (1020)

**Table 3:** Analysis of responses to comprehension questions: output from logistic and linear mixed-effects models.

	Estimate	*SE*	*z*/*t*	*p*
*Comprehension accuracy*				
Intercept	2.941	0.283	10.383	0.000*
Transitivity	0.061	0.126	0.486	0.627
Group	0.336	0.166	2.025	0.043*
Age	0.019	0.014	1.394	0.163
Transitivity × group	0.005	0.126	0.040	0.968
*Response times*				
Intercept	3.553	0.018	197.099	0.000*
Transitivity	0.006	0.005	1.260	0.219
Group	0.019	0.009	2.091	0.040*
Age	0.002	0.001	2.299	0.024*
Transitivity × group	0.002	0.003	0.550	0.582

*Effect significant at α = 0.05.

The R code for these models was as follows: ACC = glmer (Accuracy ~ 1 + Transitivity * Group + Age + (1|ITEM) + (1|SUBJECT), data = R99, family = binomial). RT = lmer (log(RT) ~ 1 + Transitivity * Group + Age + (1 + Transitivity + Group|ITEM) + (1|SUBJECT), data = R99).

Mean self-paced reading times by group and transitivity condition from the critical NP and the post-critical word (i.e., the main clause verb) can be found in Table 4. The main statistical analysis of the reading time data is reported in Table 5. At the critical region with the post-verbal NP, there was a main effect of transitivity, in which reading times were longer when the NP followed an intransitive verb versus a transitive one, and a main effect of group, with the reading times of heritage speakers being generally longer than those of the comparison group. There was no effect of age. There was no interaction of transitivity with group, which indicates that the transitivity effect was similar across both groups.

**Table 4:** Trimmed response times (SDs in parenthesis).

	Heritage speakers	Comparison group
*Critical NP*		
Transitive	866 (482)	748 (386)
Intransitive	987 (583)	868 (498)
*Critical NP + 1*		
Transitive	773 (366)	725 (350)
Intransitive	875 (599)	713 (396)

**Table 5:** Analysis of self-paced reading times: output from linear mixed-effects models.

	Estimate	*SE*	*t*	*p*
*Critical NP*				
Intercept	2.883	0.025	117.564	0.000*
Transitivity	0.025	0.007	3.422	0.002*
Group	0.032	0.015	2.234	0.029*
Age	0.002	0.001	1.665	0.100
Transitivity × group	0.002	0.006	0.257	0.798
*Critical NP + 1*				
Intercept	2.836	0.021	134.915	0.000*
Transitivity	0.006	0.006	0.995	0.330
Group	0.032	0.014	2.331	0.023*
Age	0.004	0.001	2.929	0.005*
Transitivity × group	0.011	0.005	2.218	0.027*

*Effect significant at α = 0.05.

The R code for these models was as follows: NP = lmer (log(RT) ~ 1 + Transitivity * Group + Age + (1 + Transitivity|ITEM) + (1 + Transitivity|SUBJECT), data = R3). NP + 1 = lmer (log(RT) ~ 1 + Transitivity * Group + Age + (1 + Transitivity + Group|ITEM) + (1|SUBJECT), data = R4).

At the post-critical word, the main clause verb that followed the critical NP, there was no main effect of transitivity, but there was a main effect of group, in which the reading times of heritage speakers were generally longer than those of the comparison group and there was a main effect of age, in which the reading times of older participants were also generally longer. Most importantly, transitivity interacted with group. Pairwise comparisons conducted to probe the interaction revealed that the effect of transitivity was significant for the heritage group, estimate = 0.033, *SE* = 0.012, *t* = 2.841, *p* = 0.009, but not for the comparison group, estimate = 0.009, *SE* = 0.019, *t* = 0.483, *p* = 0.630.

The transitivity effect can be taken as a sign of efficient online processing across both groups, but the spillover effect that was evident only among the heritage speakers might be related to any of several language background variables that differed both between the two groups and especially among the heritage speakers. For this reason, we conducted a secondary set of statistical analyses to explore what language background and reading-based variables might play a role in this aspect of sentence processing among heritage speakers. Each model examined the effect of transitivity, one centered background variable (run separately to avoid issues with multicollinearity), and their interaction. Age was again included as a covariate. As with the main analyses above, each model had random intercepts for subject and item and random slopes for transitivity by subject and by item wherever possible (i.e., as with the main analyses above, the slopes were simplified if the model did not converge). A total of five background variables from Table 1 were analyzed for both the critical noun phrase and the post-critical region: age of acquisition of English, DELE proficiency test score, self-reported current exposure to Spanish, self-rated reading ability in Spanish, and average reading speed for the self-paced reading task (calculated as the mean reading time across all sentence regions and across all sentences in the self-paced reading task, including experimental items, distractors, and fillers).

All ten models showed a main effect of transitivity (*t*s > 2.3 and *p*s < 0.03) and most also showed a significant or marginally significant effect of age, consistent with the main analyses above. There were also main effects at both stimulus regions for the DELE proficiency test score (R3: estimate = 0.006, *SE* = 0.002, *t* = 3.436, *p* = 0.001; R4: estimate = 0.006, *SE* = 0.002, *t* = 3.498, *p* = 0.001), for self-rated reading ability in Spanish (R3: estimate = 0.018, *SE* = 0.006, *t* = 2.944, *p* = 0.005; R4: estimate = 0.024, *SE* = 0.006, *t* = 4.031, *p* < 0.001), and average reading speed (R3: estimate = 0.000, *SE* = 0.000, *t* = 16.752, *p* < 0.001; R4: estimate = 0.000, *SE* = 0.000, *t* = 13.014, *p* < 0.001), but not for age of acquisition of English or self-reported current exposure to Spanish (all *t*s < 0.90 and *p*s > 0.40). The main effects reflected generally longer reading times with a lower DELE score, with a lower self-rating for reading ability, and with slower average reading speed. The interaction with transitivity was significant only at the critical NP and only in the models with self-rated reading ability in Spanish (R3: estimate = 0.008, *SE* = 0.003, *t* = 3.041, *p* = 0.004; R4: estimate = 0.003, *SE* = 0.002, *t* = 1.382, *p* = 0.167) and average reading speed (R3: estimate = 0.000, *SE* = 0.000, *t* = 3.962, *p* < 0.001; R4: estimate = 0.000, *SE* = 0.000, *t* = 0.744, *p* = 0.457); other *t*s < 1.4 and *p*s > 0.15. These interactions reflected a more pronounced transitivity effect at the critical NP with lower self-ratings for reading and with slower average reading speed.

Thus, the main results of this investigation can be summarized as follows:

- The expected main effect of transitivity was evident on the critical NP across both groups: reading times were longer when the NP followed an intransitive verb than when it followed a transitive verb.
- The same effect spilled over to the post-critical region, the main clause verb, but only among the heritage speakers.
- Additional analysis of the heritage speaker data with language background variables revealed that greater transitivity effects were associated with lower self-ratings for reading and with slower average reading speed, but this was only on the critical NP and not on the spillover region.
- The heritage speakers also showed generally longer reading times for the stimulus sentences and longer response times and lower accuracy for the post-stimulus comprehension questions than the comparison group.

## 5. Discussion

The present study examined the processing of verb transitivity among heritage speakers of Spanish and a comparison group of more prototypical L1 users who had acquired Spanish in a majority language context before immigrating to the U.S. as adults. Both groups showed the expected effect during self-paced reading, which suggests that they successfully integrated verb transitivity specifications and the structural principle of Late Closure during online processing, as it is the conflict between the two that is thought to underlie the processing effect in question. This outcome was tentatively predicted based on previous research that had observed the same processing effect with other populations of early bilinguals (Berghoff, 2020; McCormick, 2020) and with adult L2 learners with advanced proficiency (Roberts and Felser, 2011; Jegerski, 2012). Thus, there is now a growing body of evidence that shows that a range of bilingual language users are sensitive to verb transitivity during processing, although it should be noted that some groups of L2 participants have failed to show the online effects in question (Frenck-Mestre and Pynte, 1997; McCormick, 2020; Brothers et al., 2021), probably due to having a lower level of proficiency. This observation also suggests that the processing of verb argument specifications may be an area of so-called “resilience” among heritage speakers, which is an important gap in the knowledge base noted by Polinsky and Scontras (2020).

In addition to the basic effect that occurred at the critical region of the stimulus sentences (i.e., the post-verbal noun phrase), the heritage speakers displayed a continued effect that carried over into the following region. This apparent spillover effect was not evident among the comparison group in the present study, nor was it observed among any of the participant groups in two previous studies with very similar stimuli that also manipulated verb transitivity (Jegerski, 2012; McCormick, 2020).

On the other hand, two prior investigations of similar processing effects with stimuli that manipulated noun plausibility (as a direct object of the verb that preceded it) rather than verb transitivity had observed some type of continuation of processing effects among other participant populations. Specifically, the L2 participants in Roberts and Felser (2011) displayed longer reading times on both the critical noun and the verb that followed it, similar to the heritage speakers in the present study. An L1 comparison group in Roberts and Felser (2011) failed to show the effect on either stimulus region. In addition, Berghoff (2020) observed a prolonged processing effect among a childhood L2 group, with longer reading times on two post-critical words (and no effect on the critical noun itself). The L1 group in that study also showed a prolonged effect over the same two stimulus regions as did the L2 group, although the numerically longer reading times were only marginally significant on the second post-critical word with the L1 group. In both studies, the results were taken as evidence of greater sensitivity to semantic information such as plausibility in L2 processing as compared to L1 processing, in line with the Shallow Structure Hypothesis (Clahsen and Felser, 2006), which claims that L2 processing is more sensitive to semantic information because it can help to compensate for purported deficiencies in syntactic processing.

Thus, one explanation for the extended effect observed among the heritage speakers in the present study is that they were more sensitive to verb transitivity than the comparison group, perhaps because of a need to compensate for a lack of grammatical detail in processing, in line with the claims of the Shallow Structure Hypothesis for L2 processing (Clahsen and Felser, 2006). However, one potential problem with this account is that it is not clear to what extent the semantic plausibility effect from these previous studies is comparable to the verb transitivity effect in the present and two previous studies (Jegerski, 2012; McCormick, 2020). There is evidence that verb subcategorization specifications may be of higher priority than the semantic plausibility of nouns as objects, at least in monolingual L1 processing (Garnsey et al., 1997), which is in line with the observation from previous research that online effects appear to have been more consistent and localized with verb transitivity (Jegerski, 2012; McCormick, 2020) than with plausibility (Roberts and Felser, 2011; Berghoff, 2020). Still, verb transitivity is similar to semantic plausibility in terms of where it fits in the Shallow Structure Hypothesis, meaning it would be intact or even over-emphasized during so-called “shallow” processing (Clahsen and Felser, 2006, p. 18).

A second explanation for the extended effect observed among the heritage speakers in the present study is that it is related to reading skill. Literacy skills are typically underdeveloped in heritage speakers (Carreira and Kagan, 2011) and one prior study of heritage speakers using self-paced reading found that sentence processing was related to reading (Keating et al., 2016). Specifically, the participants who read in Spanish more often were more similar to a monolingual comparison group in their processing of pronominal reference. Along these same lines, the analysis of individual difference variables in the present study showed that greater transitivity effects were associated with slower average reading speed during the self-paced reading task and also with lower self-ratings for reading ability in Spanish. In other words, slower and less skilled readers had a larger reading time effect (i.e., greater processing difficulty) upon encountering a noun phrase that followed an intransitive verb versus a transitive one. This outcome is broadly consistent with the results of two prior investigations, albeit with different participant populations. As mentioned above, the two previous studies employed slightly different types of stimulus sentences, with those of Jegerski (2012) very closely resembling the verb transitivity stimuli from the present study and those of Roberts and Felser (2011) instead manipulating the semantic plausibility of a post-verbal noun phrase. Jegerski (2012) subdivided monolingual L1 and very advanced L2 participant groups based on a median split for average reading speed (during the experimental self-paced reading task, as in the present study) and found that only the slower L1 readers showed the processing effect in question. Reading speed did not appear to matter for the L2 group in that study, which showed the effect regardless of sub-group. Roberts and Felser (2011) performed a similar analysis and observed that slower L2 readers exhibited an extended processing effect over two stimulus regions, whereas the faster L2 readers showed the effect only on the second stimulus region. In that study, reading speed did not seem to matter for the L1 group.

It is interesting to note that both L1 and L2 processing can vary according to reading speed, but do not seem to do so consistently (i.e., across both studies). Most relevant to the present study is that greater processing difficulty on a post-verbal noun that cannot be integrated as an object of the verb immediately before it does not appear to be unique to heritage speakers. Nevertheless, to the extent that they are generally slower and less skilled readers in the heritage language, heritage speakers could potentially be more susceptible to such effects than other participant populations such as L2 and monolingual L1 users.

A third consideration in the interpretation of the extended reading time effect observed among the heritage speakers in the present study is the self-paced reading method that was employed to measure language processing. Self-paced reading appears to be particularly conducive to delayed or *spillover* effects (Just et al., 1982; Frank et al., 2013), in which a reading time difference caused by a critical word or phrase in the stimuli carries over to the following word or phrase. One reason why spillover effects might be especially common is that self-paced reading does not allow participants to reread prior text, which is very much a part of normal reading. Moreover, heritage speakers can show generally higher rates of rereading, or regressive eye movements, than monolingual L1 users (Parshina et al., 2021), so they may be more affected by self-paced reading during language processing experiments.

As outlined in the literature review sections of this paper, several previous studies have used the self-paced reading method with heritage speakers. Some of these segmented the stimulus sentences in a way that did not yield detailed enough data to observe spillover (Jegerski et al., 2016; Keating et al., 2016) or did not observe any online effects with the potential for spillover (Jegerski, 2018b), but both of the prior investigations that were able to gage spillover reported extended reading time effects that occurred on both the critical stimulus region and the following region. In one case, this was with phrase-by-phrase self-paced reading (Jegerski, 2018c), as in the present study, and in the other it was with word-by-word self-paced reading (Foote, 2011). In both cases, the same prolonged effect was displayed by a comparison group of monolinguals (Jegerski, 2018c) or of more prototypical L1 users raised in a majority language context (Foote, 2011). Thus, previous research offers no particular evidence either for or against the supposition that heritage speakers are especially likely to show spillover effects during self-paced reading, although it does serve as a reminder that such effects are common in general, not just with heritage speakers. The present study appears to be the first with heritage speakers in which the comparison group has not shown spillover, which is the best scenario for testing whether heritage speakers are more likely to show such effects.

Looking to the future, it is clear that there is a need for more research using self-paced reading with heritage speakers, in line with the broader motivation for the use of online methods laid out in the introduction and literature review sections of this article. In addition, the present study has suggested that heritage speakers may be especially likely to show spillover effects with self-paced reading, but further research using the method is needed to determine to what extent the findings of this single study may generalize to other samples of heritage speakers and other aspects of sentence processing. In addition, a follow-up study using eyetracking, which is already in progress, could help clarify to what extent shallow processing (Clahsen and Felser, 2006) may underlie the observations of the present study. Specifically, evidence from eyetracking could help tease apart shallow processing from the self-paced reading method, as an effect caused by shallow processing should hold even if the experimental method is changed to eyetracking, whereas an artifact of the self-paced reading method should not.

In conclusion, a primary finding of the present study was that heritage speakers of Spanish exhibited prolonged effects for verb transitivity across two stimulus regions during self-paced reading, whereas a comparison group of more prototypical L1 users raised with Spanish as a majority language displayed the effect only on the immediate region, with no spillover. Analysis of individual background variables revealed that reading-related metrics predicted the degree of sensitivity to verb transitivity. Three explanations for the apparent susceptibility to spillover effects among heritage speakers were proposed: that it is a characteristic of shallow processing (Clahsen and Felser, 2006), that it is due to underdeveloped reading skill (i.e., reading speed, more frequent rereading, and other skills that form the basis for self-ratings), and that it is an artifact of the self-paced reading method. The latter two possibilities are especially consistent with a role for reading skill in these results, although the three explanations are not mutually exclusive, so they might all apply to varying degrees or in different contexts.

## Data availability statement

The raw data supporting the conclusions of this article will be made available by the authors, without undue reservation.

## Ethics statement

The studies involving human participants were reviewed and approved by the Texas Tech University Protection of Human Subjects Committee. The participants provided their written informed consent to participate in this study.

## Author contributions

Both authors made an equal contribution to this work.

## Conflict of interest

The authors declare that the research was conducted in the absence of any commercial or financial relationships that could be construed as a potential conflict of interest.

## Publisher’s note

All claims expressed in this article are solely those of the authors and do not necessarily represent those of their affiliated organizations, or those of the publisher, the editors and the reviewers. Any product that may be evaluated in this article, or claim that may be made by its manufacturer, is not guaranteed or endorsed by the publisher.

## Footnotes

^1^The flanker task (Eriksen and Eriksen, 1974) is widely used as a measure of inhibitory control. In this technique, a target stimulus is flanked by non-target stimuli that can be congruent, non-congruent, or neutral. For instance, participants may be asked to indicate whether the center arrow in a row of seven is pointing to the left or right and the three arrows appearing on either side of the target might be pointing in the same direction as the target or in the opposite direction. The presence of non-congruent stimuli is typically associated with slower and less accurate responses, so a left-pointing arrow is harder to identify when it is flanked by right-pointing arrows than by left-pointing ones.
